# Craving on the move: targeting smoking memories with a novel 3MDR–smoking cessation protocol

**DOI:** 10.3389/fpsyt.2024.1398027

**Published:** 2024-12-10

**Authors:** Annel P. Koomen, Anne Marije Kaag, Kees A. M. Jongenelen, Rianne Wouters, Hein J. F. van Marle, Odile A. van den Heuvel, Ysbrand D. van der Werf, Taco J. De Vries

**Affiliations:** ^1^ Department of Anatomy and Neurosciences, Amsterdam University Medical Center, Amsterdam, Netherlands; ^2^ Department of Clinical Neuro and Developmental Psychology, Vrije Universiteit Amsterdam, Amsterdam, Netherlands; ^3^ Department of Psychiatry, Amsterdam University Medical Center, Amsterdam, Netherlands; ^4^ GGZ inGeest Mental Health Care, Amsterdam, Netherlands; ^5^ Mood, Anxiety, Psychosis, Stress and Sleep, Amsterdam Neuroscience, Amsterdam, Netherlands; ^6^ ARQ National Psychotrauma Center, Diemen, Netherlands; ^7^ Compulsivity Impulsivity and Attention, Amsterdam Neuroscience, Amsterdam, Netherlands

**Keywords:** tobacco use disorder, smoking, craving, EMDR, smoking cessation, cue exposure, memory reconsolidation, working memory

## Abstract

**Introduction:**

Improved effectiveness and treatment adherence is needed in smoking cessation (SC) therapies. Another important challenge is to disrupt maladaptive drug-related memories. To achieve these goals, we developed a novel treatment strategy on the basis of motion-assisted memory desensitization and reprocessing (3MDR).

**Methods:**

In this study, the added effect of a distractor task following memory recall during a newly designed 3-day SC version of 3MDR (3MDR-SC) protocol on reducing smoking cue–elicited craving was investigated in abstinent chronic smokers. Chronic smokers were randomly allocated to an active 3MDR-SC group (receiving 3MDR-SC with a working memory distractor task) (*n* = 42) or a control 3MDR-SC group (receiving 3MDR-SC with a non-distracting task) (*n* = 39). Smoking cue–induced craving and physiological measures were assessed at baseline (T0) and 1 day after the intervention (T4), and smoking behavior was measured at T0 and 2-week (FU1) and 3-month (FU2) follow-up.

**Results:**

Significant decreases in cue-induced craving from T0 to T4 and daily cigarette use from T0 to FU1 and FU2 were observed but not differ between the two experimental groups. Cue-induced changes in heart-rate variability and skin conductance, which did not differ from T0 to T4, and relapse at FU2 were also not different between groups. Dropout rate during intervention was 2.5%.

**Conclusions:**

The 3-day 3MDR-SC intervention resulted in a reduction in cue-induced craving and smoking behavior and showed very good treatment adherence. There was no added effect of the distractor task on 3MDR-SC efficacy. Further studies, including a treatment as usual control, are needed to confirm 3MDR-SC as an effective SC therapy.

## Introduction

As smoking-related annual deaths amount to 8 million worldwide (1), smoking is the largest preventable health risk and the leading cause of premature deaths (2). The risk of relapse of 92% for nicotine without cessation support 3 months after a quit attempt (3) is higher than for other addictive substances (4). Currently, the most effective smoking cessation (SC) therapy is a combination of behavioral and pharmacological therapy (5), resulting in long-term abstinence rates of 15% (6) and high dropout rates of up to 77% (7). The large time investment of behavioral therapies and adverse side effects of SC medication (8) might all contribute to these high attrition rates. Thus, improved effectiveness and treatment adherence is needed in SC therapies.

An important challenge in addiction treatment is to disrupt maladaptive drug-related memories (9) that, once activated by environmental cues, elicit drug craving and promote drug use (9, 10). Repeated exposure of drug cues as in cue-exposure therapy (CET) is a way to extinguish drug-related memories (11). However, the high context specificity of the therapy (12) limits the clinical effects of CET in addiction, including tobacco use disorder (13). Adding virtual reality in CET (VR-CET) for SC further improves ecological validity by exposing smokers to a fuller range of cigarette-related environmental stimuli as compared to more traditional CET methods (14) and has shown promising clinical results on craving and smoking behavior (15, 16). Unfortunately, high dropout rates (22%) following 8-week VR-CET were observed, probably due to strong intervention-induced craving and withdrawal symptoms (16). Shorter SC interventions with a faster reduction in craving in abstinent smokers might, therefore, result in better attrition rates.

To blunt drug-related memories, a more promising approach focuses on disruption of memory reconsolidation (9, 50). When tasks that tax working memory, such as performing eye movements, are performed during reconsolidation of retrieved emotional memories, reduced vividness of these memories is experienced (17). Eye movement desensitization and reprocessing (EMDR) is based on this principle and is an effective treatment for post-traumatic stress disorder (PTSD; 17). Elevation of working memory load after retrieval of smoking memories was found to reduce cigarette craving in abstinent smokers (18, 19). However, whether EMDR might be an effective treatment for tobacco addiction remains unclear. Furthermore, EMDR protocols, so far, used conditioned stimuli to recall addiction memories, whereas both human and rodent studies showed stronger desensitization when addiction memories were also retrieved by the unconditioned stimulus, i.e., the drug itself (20).

Motion-assisted memory desensitization and reprocessing (3MDR) is a novel treatment that combines EMDR, VR-CET, and movement (21) to increase treatment efficacy (22). During 3MDR, patients walk through a virtual environment toward personalized stimuli to retrieve emotional memories, followed by a working memory distractor task to reduce the emotionality of these memories (21). A reduction in clinical symptoms was observed after 3MDR in patients with PTSD (23), which was highest for 3MDR than other VR-CET combined interventions (24). Furthermore, a reduction in PTSD symptoms after 3MDR was only observed when memory recall was followed by a distractor task (an eye movement task) as compared to that by no distractor task (25). To date, the effectiveness of 3MDR and added effect of a distractor task has not yet been investigated in the treatment of tobacco use disorder.

The aim of the current experimental lab study was to develop and test a SC version of a 3MDR protocol (3MDR-SC). An active control group was used as comparison. This group did not receive a working memory distractor task following the recall of smoking memories. In short, abstinent chronic smokers were randomly allocated to a group receiving 3MDR-SC using a working memory distractor task following memory recall (active 3MDR-SC group) or a group receiving 3MDR-SC using a non-distracting task (control 3MDR-SC group). In this protocol, not only conditioned but also unconditioned nicotine stimuli (two puffs of a cigarette) were used to reactivate addiction memories. It was hypothesized that, in the distractor group as compared to that in the non-distractor 3MDR-SC group, there would be a greater decline in cue-induced craving (primary outcome) and physiological measures including heart-rate variability (HRV) and skin conductance (secondary outcome) from baseline to the end of the 3MDR-SC intervention and greater decline in smoking behavior (secondary outcomes) from baseline to 2-week and 3-month follow-up (nicotine use severity and daily cigarette use) and at follow-up (relapse).

## Materials and methods

The present study was approved by the medical ethics committee of the *Vrije Universiteit Medisch Centrum* and pre-registered in the Dutch Trial Register (https://clinicaltrialregister.nl/en/trial/27225). Written informed consent was provided by all participants before study participation in accordance with the Declaration of Helsinki. Participants received a monetary reward for each attended study session.

### Participants

Chronic smokers (minimum of 10 cigarettes per day for a minimum of 10 years, and age between 25 and 55) were recruited via social media and local advertisements in the Amsterdam area, The Netherlands. Exclusion criteria were a *1)* neurological disorder, *2)* lifetime diagnosis of or treatment for psychosis or mania, *3)* other psychiatric diagnosis or treatment in the past year, *4)* current use of psychotropic drugs, *5)* current substance dependence other than nicotine, indicated by an Alcohol Use Disorders Identification Test (AUDIT; 26) and Drug Use Disorders Identification Test (27) scores of higher than 12, *6)* mobile impairment, and *7)* inability to understand study procedures.

### General study procedures

Individuals interested in study participation received an online screening form, assessing the inclusion and exclusion criteria.

The study consisted of five consecutive measurements (see **Supplementary Figure S1** for an overview of study procedures), including a baseline measurements (T0); a 3-day 3MDR-SC intervention T1, T2, and T3; and post-intervention measurements (T4). Participants were asked to stop consuming cigarettes or other nicotine-containing substances 48 h before the start of T0 and to remain abstinent throughout all five sessions.

Smoking abstinence was monitored at the start of T0–T4 by measuring exhaled carbon monoxide levels with calibrated Smokerlyzer (Micro+) breath tests, and non-abstinent (>10 ppm) participants were excluded from further study participation. Participants were randomly allocated to the distractor or non-distractor 3MDR-SC intervention group with sex as a stratification factor. Participants were blinded to the assigned condition. Follow-up assessments were done via online questionnaires 2 weeks (FU1) and 3 months (FU2) after T4.

### Cue-exposure task

The computer-assisted cue-exposure task was performed by the participants at T0 and T4. The task started and ended with the presentation of the 10 items from the brief Questionnaire on Smoking Urges (QSU-brief) to measure the state-dependent urge to smoke, distinguishing between reward- and relief-related craving (28). In between, participants were exposed to visual smoking cues (videos and pictures) and handling of smoking paraphernalia. A detailed description of the cue-exposure task can be found in the **Supplementary Methods** and **Supplementary Table S1**.

### HRV and skin conductance measurements

Physiological parameters, HRV (29, 30) and skin conductance (31, 32), were measured continuously throughout the cue-exposure task and 3MDR-SC treatment with the Vrije Universiteit Ambulatory Monitoring System (VU-AMS; 33). Details of the HRV and conductance measurements and processing steps are described in the **Supplementary Material**.

### Personalized picture craving ratings

At T0 and T4, cigarette craving in response to 10 self-chosen personal pictures strongly associated with their smoking behavior was assessed using a 10-point Likert scale. The six pictures showing the highest craving ratings at T0 were selected for the 3MDR-SC treatment, with two pictures per treatment.

### Questionnaires

After T0, several online questionnaires were sent to the participants to assess baseline characteristics. The Fagerström Test for Nicotine Dependence (FTND) was used to assess nicotine dependence severity over the last year (34), and the Timeline Follow-Back method (TLFB) for nicotine was used to calculate mean daily cigarette use as measured by self-reported smoked cigarettes in the last 14 days (minus the two required abstinence days at T0) (35).

At FU1 and FU2, participants received a link to online questionnaires to measure nicotine dependence severity with the FTND and daily cigarette use and relapse using the TLFB over the last 14 days.

Other questionnaires filled out by the participants are described in the **Supplementary Material**.

### 3MDR-SC treatment

**Figure 1:**
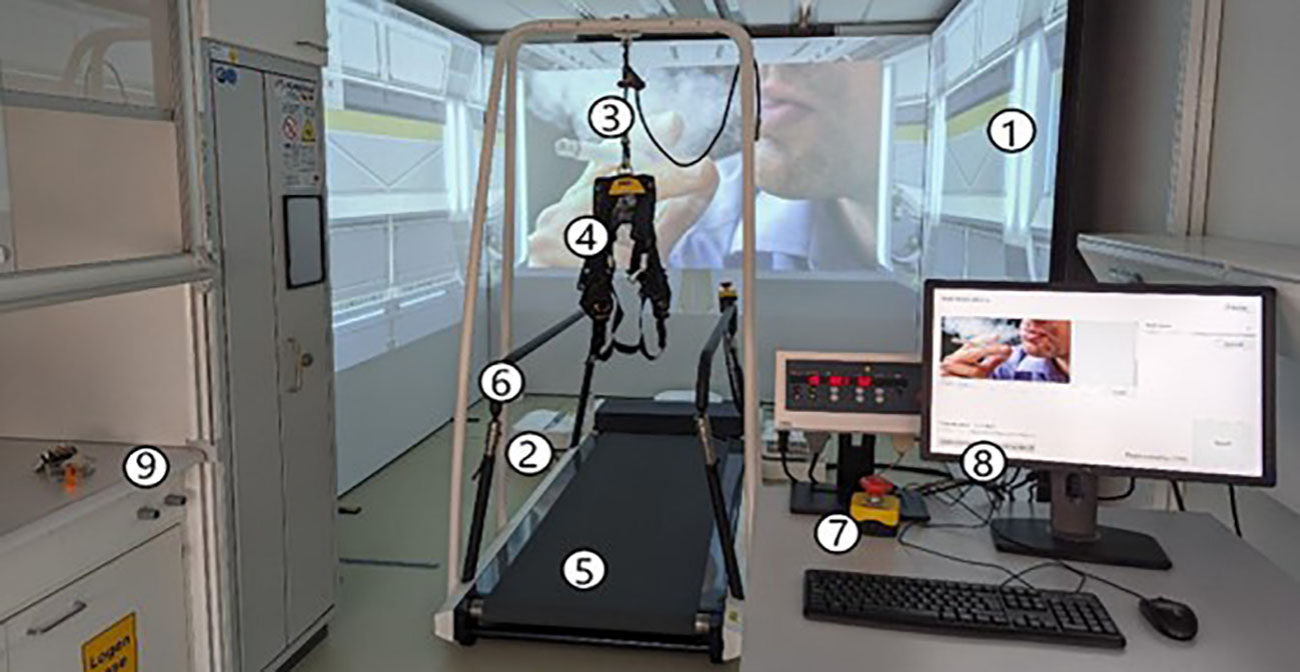
3MDR-SC system setup. Shown are the projection screens (1), projectors (2), safety line (3) for safety harness (4), treadmill (5), safety handrails (6), emergency stop button (7), computer with D-Flow 3MDR software (8), and fume hood (9).

Participants underwent a 3-day 3MDR-SC treatment at T1, T2, and T3. The 3MDR-SC system setup by Motekforce Link included a treadmill, projection screens, safety equipment, and D-Flow software (**Figure 1**). 3MDR-SC treatment involved recall of smoking-related memories by smoking and smoking cues (two cigarette puffs and two self-chosen pictures), which were then followed by a working memory distractor task (active 3MDR-SC) or non-distractor task (control 3MDR-SC). Current cigarette craving was assessed on a scale from 1 to 10 when in motion at baseline, during memory recall before and after the (non-)distractor task. For a more detailed description, see **Supplementary Material**.

### Statistical analysis

A detailed description of all statistical analyses performed is provided in the **Supplementary Material**.

### Main and secondary outcome analyses

Repeated-measures analyses of variance (rmANOVAs) were performed to investigate whether changes in cue-induced craving (difference QSU-brief sum score following cue exposure) over sessions (T0 and T4) and nicotine use severity and daily cigarette use (mean TLFB-nicotine amount) over time (T0, FU1, and FU2) were moderated by treatment group (active and control 3MDR-SC). A linear mixed model with random intercept was performed to test whether changes in HRV [root mean square of successive differences (RMSSD)] and skin conductance [mean skin conductance level (SCL)] during the nicotine cue-exposure task blocks (relax, video, picture, and handling) over sessions (T0 and T4) were moderated by treatment group. Group differences in relapse at FU2 (not-even-a-puff criterion assessed with TLFB-nicotine) were assessed with chi-square tests. Participants lost to follow-up were considered as being relapsed, and relapse over the last 7 (36) and 14 days was tested.

### Exploratory analyses

rmANOVAs were performed to investigate whether changes in craving for personalized pictures over sessions (T0 and T4) per picture type (selected and non-selected during treatment) and craving following memory recall over the treatment sessions (T1, T2, and T3) at every treatment phase (at baseline and before and after task for three cues) were moderated by treatment group.

## Results

A flowchart of all individuals involved in the study is provided in **Supplementary Figure S2**. Of all scheduled participants, 84.9% received at least one 3MDR-SC treatment session (and 82.8% all three treatment sessions). Moreover, 6.0% of the enrolled participants dropped out of treatment (2.5% after receiving at least one 3MDR-SC session). Lost–to–follow-up rates (at FU1 and FU2) were 12.0% for enrolled participants (and 8.9% after receiving at least one 3MDR-SC session).

### Baseline characteristics

On average, the 81 participants had an age of 41.7, had been using cigarettes for a duration of 26.8 years, and smoked 14.5 cigarettes per day. There were no significant differences for any of the sociodemographic, nicotine-related, clinical, and study-related characteristics at baseline present between the participants in the active 3MDR-SC (*n* = 42) and control 3MDR-SC (*n* = 39) groups (see **Supplementary Table S2**).

### Main and secondary outcomes

#### Cue-induced craving at T0 and T4

**Figure 2:**
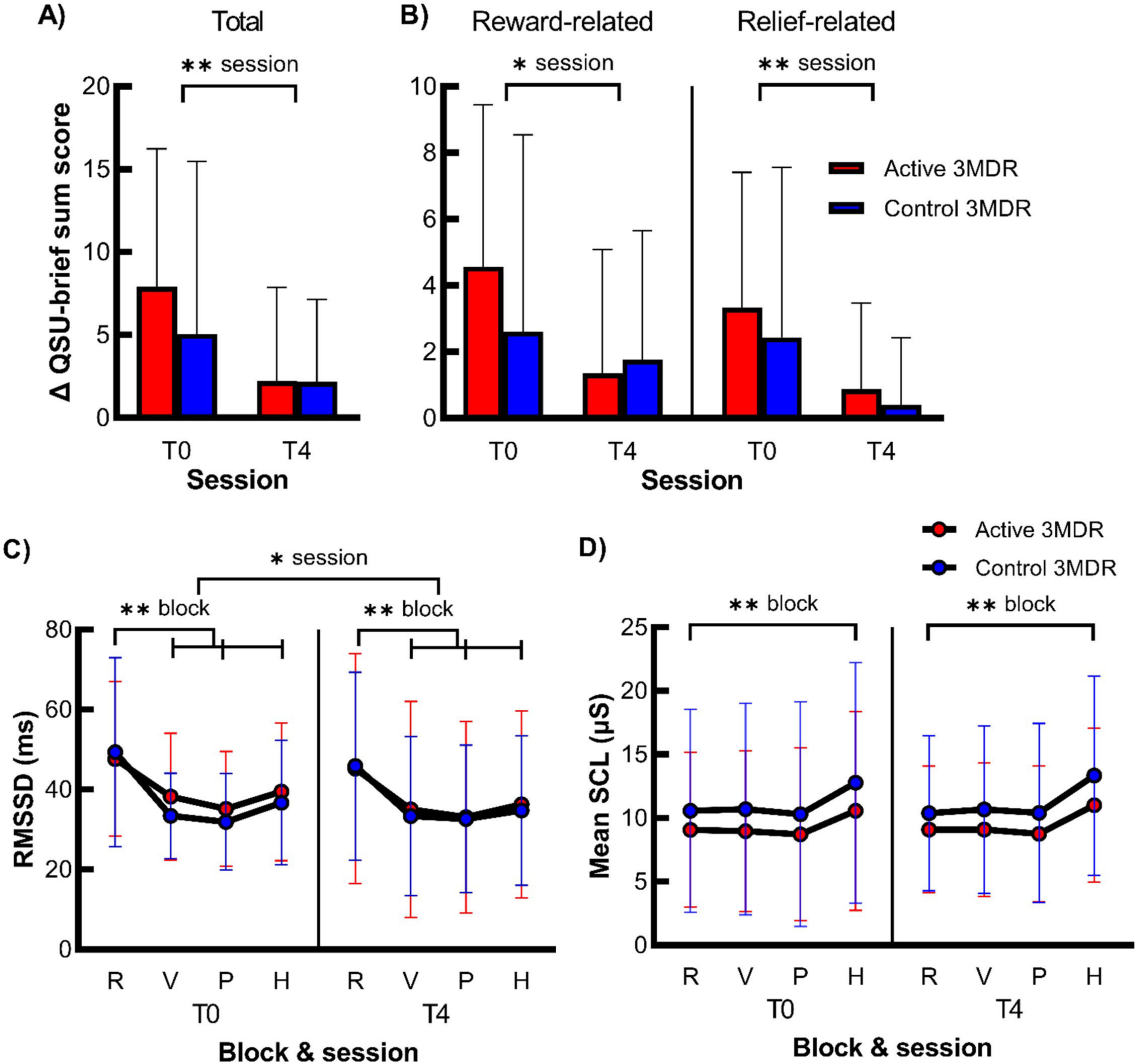
The effect of active 3MDR-SC on cue-induced craving and cue-reactivity. Shown is the mean (+ SD) difference in QSU-brief sum score after cue exposure for all items **(A)** and reward- and relief-related craving items **(B)** (*n* = 75) and of the mean (± SD) RMSSD **(C)** and mean SCL **(D)** during the blocks relaxation (R), video (V), picture (P), and handling (H) blocks of the nicotine cue-exposure task (*n* = 81) at session T0 and T4 in the active 3MDR-SC (red) and control 3MDR-SC (blue) groups. * session, main effect of session of p < 0.05. ** session, main effect of session of p < 0.001. ** block, main effect of block of p < 0.001. R, relaxation; V, video; P, picture; H, handling. Of the HRV data, 10.6% of data points were missing (*n* = 69), and of the skin conductance data, 6.9% of the data points were missing (*n* = 45).

Cue exposure resulted in increased craving, as represented by a significant moderate increase in the QSU-brief sum score at T0 after cue exposure (Mdn = 38) as compared to that before (Mdn = 29; *Z* = −5.506, *p* < 0.001, *r* = −0.433). For the total score and the reward- and relief-related factors of the QSU-brief separately, no significant session (T0 and T4) * group (active and control) interaction was present. Independent of treatment group, there was a main effect of session, with a strong significant reduction in cue-induced craving from T0 to T4 for total QSU-brief sum score, medium reduction in reward-related QSU-brief sum score, and large reduction in relief-related QSU-brief sum score. See **Figure 2** and **Supplementary Table S3** for all statistics.

#### Physiological measures at T0 and T4

Cue exposure resulted in physiological reactivity, as main effects of task block and pairwise comparisons revealed a large decrease in HRV during cue exposure and large increase in skin conductance during the handling cue-exposure block at T0. However, no significant three- or two-way interaction between session (T0 and T4) * task block (relax, video, picture, and handling) * group (active and control) was found on HRV and skin conductance. There was a small main effect of session on HRV and pairwise comparisons revealed that HRV was higher at T0 than T4, independent of task block and treatment group. Moreover, there was a large main effect of block on HRV and medium effect of block on skin conductance. Again, pairwise comparisons revealed lower HRV during all cue-exposure task blocks (video, picture, and handling) than relaxation and higher conductance during the handling cue-exposure task block than relaxation, independent of session and treatment group. For all statistics, see **Figure 2** and **Supplementary Table S4**.

#### Smoking behavior

**Figure 3:**
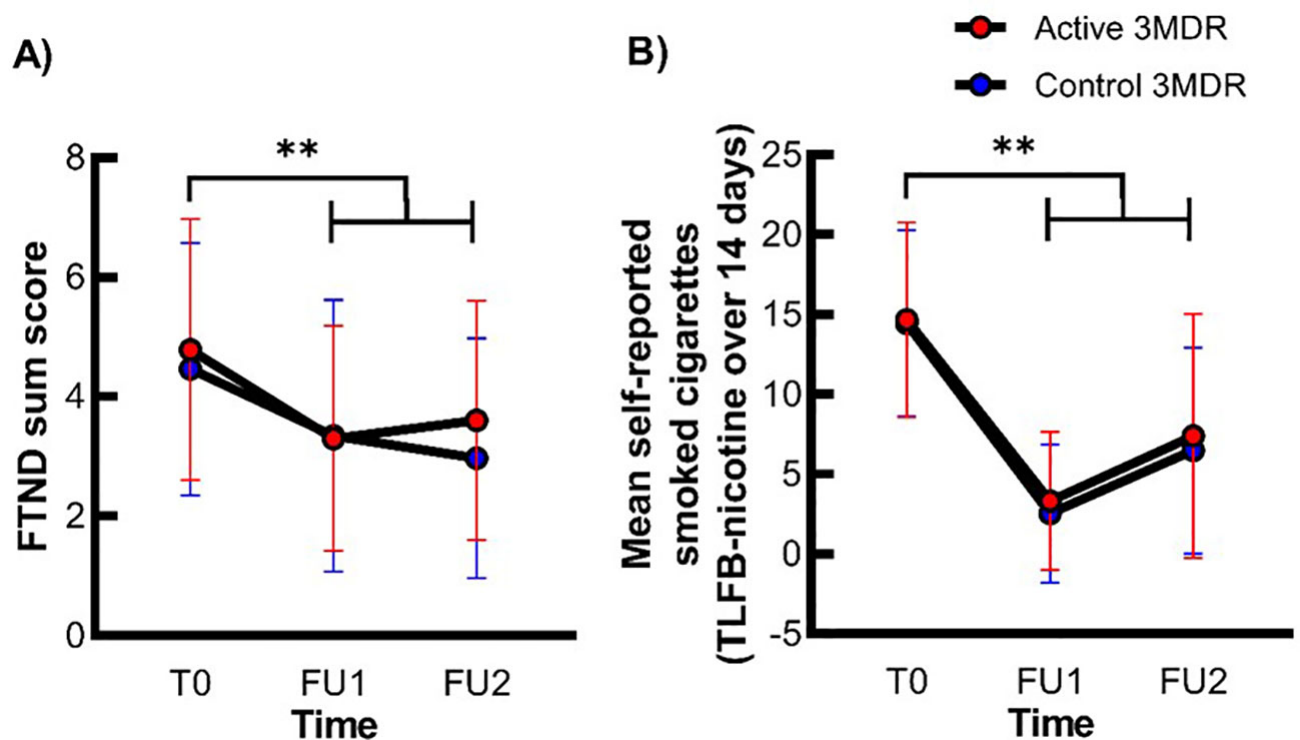
The effect of active 3MDR-SC on nicotine dependence severity and daily cigarette use at follow-up. Shown is the mean (± SD) FTND sum score **(A)** and mean self-reported smoked cigarettes **(B)** at session T0, FU1, and FU2 in the active 3MDR-SC group (red) and the control 3MDR-SC (blue) group (*n* = 71). **, main effect of time of p < 0.001.

For the FTND sum score and daily cigarette use, a non-significant interaction between time (T0, FU1, and FU2) * group (active and control), but a significant large main effect of time was present (see **Figure 3**, **Supplementary Table S5**). Simple contrasts revealed a significant reduction in FTND sum score and daily cigarette use from T0 to FU1 and from T0 to FU2, independent of treatment group. These effects were independent of group. For all statistics, see **Figure 3** and **Supplementary Table S5**.

#### Relapse

The relapse rate over the last 14 days of 70% (*n* = 28) in the active group was not significantly different from the relapse rate of 77.8% (*n* = 28) in the control group (*χ* ^21^ = 0.591, *p* = 0.442). No significant group differences in relapse rate over the last 7 days of 67.5% (*n* = 27) in the active group and 72.2% (*n* = 26) in the control group were present as well (*χ* ^21^ = 0.200, *p* = 0.655).

### Exploratory analyses

#### Craving for personalized pictures

**Figure 4:**
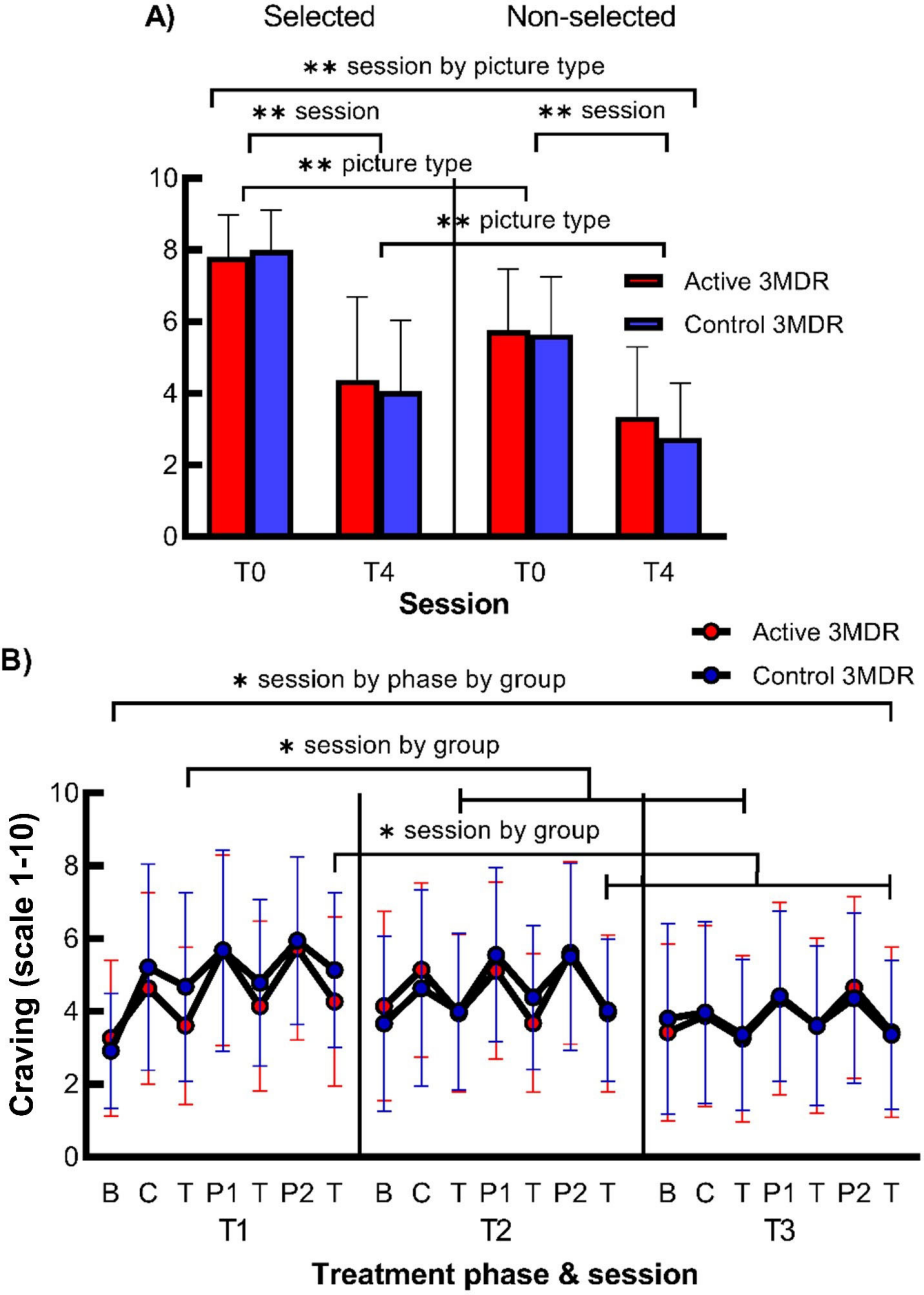
The effect of active 3MDR-SC on craving for smoking-related personalized pictures directly following treatment and on craving following memory recall of smoking-related cues during treatment. Shown is **(A)** the mean (+ SD) craving rating at session T0 and T4 for the self-chosen pictures that were selected and not-selected during treatment (*n* = 76) and **(B)** mean (± SD) craving at session T1, T2, and T3 at baseline (b) after memory recall by cigarette (c) or personal picture (P1,2) and after the (non-)distractor task (T) (*n* = 76) in the active 3MD group (red) or the control 3MDR-SC group (blue). * session by phase by group, session by treatment phase by group interaction effect of p < 0.05. * session by group, session by group interaction effect of p < 0.05. ** session by picture type, session by picture type interaction effect of p < 0.001. ** session, main effect of session of p < 0.001. ** picture type, main effect of picture type of p < 0.001. B, baseline; C, puffed cigarette; T, (non-)distractor task; P1, first personalized picture; P2, second personalized picture.

There was a significant interaction on mean craving rating for the smoking-related self-chosen pictures between session (T0 and T4) * picture type (selected and non-selected pictures), but this was not moderated by treatment group. Separate rmANOVAs per picture type showed that significant main effects of session were apparent in both selected and non-selected pictures, whereas separate rmANOVAS per session revealed that significant main effects of picture type were also apparent at both T0 and T4. As both analyses did not explain the interaction effect, this session * picture type interaction was rather interpreted as an absolute steeper decrease of craving from T0 to T4 for selected than non-selected pictures, although, for both picture types, the decrease in craving from T0 to T4 was significant. For all statistics, see **Figure 4** and **Supplementary Table S6**.

#### Craving during the 3MDR-SC sessions

There was a significant small three-way interaction for craving between session (T1, T2, and T3) * treatment phase (baseline and following memory recall before and after task for three smoking cues) * group. Subsequent analyses were performed to investigate this three-way interaction. Separate rmANOVAs per treatment group, showing significant session * treatment phase interactions for both groups, and separate rmANOVAS per treatment and three sessions, showing significant main effects of session for all sessions per group, did not explain the observed three-way interaction effect. rmANOVAs per treatment phase (baseline and following recall before and after task for three cues), revealed a significant session * group interaction effect for craving only at the third [T(c)] and seventh [T(P2)] treatment phases. Repeated contrasts indicated that craving following memory recall of the cigarette puff cue and second picture cue was lower after the distractor task in the active 3MDR-SC group than after the non-distracting task in the control 3MDR-SC group at T1 and not at T2 and T3. See **Figure 4** and **Supplementary Table S7** for all statistics.

#### Other exploratory analyses

The results of all exploratory analyses are described in the **Supplementary Results** and did not show significant group differences. In short, craving, nicotine withdrawal symptoms, depressive symptoms, and state anxiety decreased significantly over time (from T0 to FU2) for both the active and control 3MDR-SC groups. Exploratory analyses on HRV and SCL during treatment (T1, T2, and T3) revealed that HRV and SCL were significantly lower in the active group compared to those in the control group during treatment, but this effect was only present in the first treatment session (T1).

## Discussion

The aim of this study was to evaluate the effect of a novel 3-day 3MDR-SC intervention (with and without working memory distractor) on cue-elicited craving in abstinent smokers. A significant decrease in cue-induced craving from T0 to T4, as well as reduced nicotine dependence severity and daily cigarette use from T0 to FU1 and FU2, was observed in both 3MDR-SC groups, with no differences between the treatment groups. Cue-induced changes in HRV and conductance did not differ from T0 to T4, and relapse rates at FU2 (67.5%–70% in the working memory distractor group and 72.2%–77.8% in the no distractor group) were not different. Craving evoked by the personal smoking-related pictures (selected or not during 3MDR-SC) was strongly reduced from T0 to T4. Lastly, during the 3MDR-SC session, the craving evoked by either the unconditioned stimulus or the personal pictures was reduced by both the distractor and the non-distractor task. At T1, a larger reduction in craving was observed in the distractor group. Exploratory analyses demonstrated that symptoms of anxiety, depression, and withdrawal significantly reduced from T0 to the second follow-up. In conclusion, both the distractor and non-distractor 3MDR-SC intervention effectively reduced cue-induced craving and smoking behavior in heavy smokers.

The observation that the additional working memory distractor task had no added effect is in contrast to what was expected from the EMDR principles of working memory tasks following memory recall (17) and reducing cigarette craving in preclinical work (18, 19). Recently, the importance of working memory taxing distractor tasks for 3MDR efficacy on reducing clinical symptoms of PTSD, specifically traumatic memories, was investigated in a pilot-study by Roy et al. (25). Importantly, no significant differences between 3MDR with and without distractor task were present in that study. A possible explanation is that the task performed in the control group might actually have functioned as a distractor task. That is, walking on the treadmill, the passing virtual environment and the noise of the treadmill while having to focus on a steady point forward could be distracting enough and demand working memory resources (37). Therefore, an additional distractor task might not lead to a higher treatment efficacy. Alternatively, it could be that the effect predominantly relies on CET (extinction) principles of the treatment rather than the EMDR (simultaneous working memory taxing) principles. In particular, during the later treatment sessions, the CET effects might become more pronounced (11) as opposed to immediate EMDR effects on the reduction in craving (19). This is supported by the current finding of a greater craving reduction during 3MDR-SC in the distractor group at the first treatment session. The VR used in the intervention may have promoted the CET effects on cue-induced craving (16), as well as the combination of CET with movement. Indeed, movement was found to increase therapeutic CET effects in PTSD, possibly by facilitating extinction learning (38). Moreover, explicit retrieval of memories before the extinction procedure as in the control 3MDR-SC group to disrupt rather than extinguish pathological memories might also be an important ingredient for effectively blunting these memories (39, 40). This effect seems to be enhanced when addiction memories are recalled by unconditioned stimuli (51) as in our protocol, an uncommon practice in CET and EMDR studies.

Cue-induced craving is thought to promote drug use and relapse (9). In line with this, smoking behavior (i.e., nicotine dependence severity and cigarette use) was significantly reduced from T0 to FU1 and FU2 in both 3MDR-SC groups. The 7-day relapse rates at FU2 appear lower than the 92% observed after no care (3) and more comparable to the 65% after a combination of behavioral and pharmacological therapy (41). A direct comparison with a TAU group is necessary in order to determine whether 3MDR-SC has additional value as a SC therapy.

In contrast to previously reported increases in HRV during cue-exposure (30), we demonstrated cue-induced reduction in HRV. Cue-induced HRV elevations are, however, not always detected in smokers (42), correlate inversely with the amount of problematic substance years (30), or depend on substance approach or avoidance strategies (43). Importantly, excessive HRV reactivity observed in substance abuse in response to challenges is rather described as a large HRV reduction instead of an increase (44), as in line with our results.

Generalizability of the 3MDR-SC effects on nicotine craving reduction was suggested by a large reduction in craving for personalized pictures, independent of their selection during 3MDR-SC intervention. Sustained reductions in smoking behavior, craving, withdrawal, and clinical symptoms up to 3-month follow-up as demonstrated by exploratory analyses furthermore suggest that 3MDR-SC effects might be robust against spontaneous recovery over time, although studies with longer follow-up periods are needed to confirm this.

This is the first study investigating a novel 3MDR-SC paradigm, combining EMDR and VR-CET principles, on blunting maladaptive memories in tobacco addiction. The very brief, non-invasive, emotional engaging nature of 3MDR-SC seem to contribute to the low dropout rate of 2.5%, which is impressive considering the treatment adherence problem in addiction therapies. Independent of the used task (distractor or non-distracting), this study has demonstrated that 3MDR-SC is able to show significant reductions in nicotine dependence severity, cigarette use, craving, withdrawal symptoms, depression, and anxiety symptoms up to 3 months in heavily nicotine-dependent, chronic smokers.

This study has several limitations. First, since most people relapse in the initial days following abstinence (45), the required 7-day abstinence could have prevented individuals to participate in the study, as indicated by the low rates of included participants eventually receiving treatment. Earlier research demonstrated that the number of abstinent days in the 7 days following a quit attempt significantly predicts relapse (46). As we required the participants to remain abstinent during the first 5 days of the study, this may have led to a selection bias. However, the low dropout rates of 2.5% in the current study rather advocate for 3MDR-SC treatment–specific effects. Secondly, exhaled CO was used to verify abstinence from cigarettes in our study. This method is not sensitive enough to capture use of electronic nicotine delivery systems and is not applicable to use of tobacco products that are not inhaled (e.g., snus or chewing tobacco). It is possible that some participants were not completely abstinent from nicotine during the intervention. Also, during online follow-up, there was no biological confirmation of self-reported cigarette use. This may have biased our findings. Although self-reported measures such as the time-line follow-back procedure is widely acknowledged as reliable for gathering information about substance use, including cigarette use (47, 49), in our study, no information was gathered on other nicotine containing products. Lastly, although craving for the smoking-related pictures following 3MDR-SC treatment was significantly decreased, craving induced by the pictures was not completely diminished. Thus, no complete desensitization of the personalized pictures was achieved, as recommended by previous EMDR studies (48).

Future studies should incorporate a *treatment as usual* control group to verify the clinical effects of 3MDR-SC in tobacco use disorder on smoking behavior and relapse. To avoid small attrition rates before treatment due to dealing with withdrawal symptoms, combining 3MDR-SC with nicotine replacement therapy can be considered. Spontaneous recovery can be investigated by using a follow-up period of at least 6 months and can be prevented by repeating treatment sessions after the first week if nicotine craving is still experienced, aiming for full desensitization of addiction memories.

In conclusion, the brief and newly designed 3MDR-SC intervention showed a significant reduction in cue-induced craving and smoking behavior, independent of a distractor task. Further clinical research on 3MDR-SC as SC therapy is warranted.

## Data Availability

The raw data supporting the conclusions of this article will be made available by the authors, without undue reservation.
