# Supplementary material for: Craving on the move: targeting smoking memories with a novel 3MDR–smoking cessation protocol
Source: Front Psychiatry. 2024 Dec 10;15:1398027. doi: 10.3389/fpsyt.2024.1398027 (PMC11666516; doi:10.3389/fpsyt.2024.1398027)
Supplement: Supplementary file 1 [file DataSheet1.pdf]

![frontiers logo]

Supplementary Material

Supplementary methods

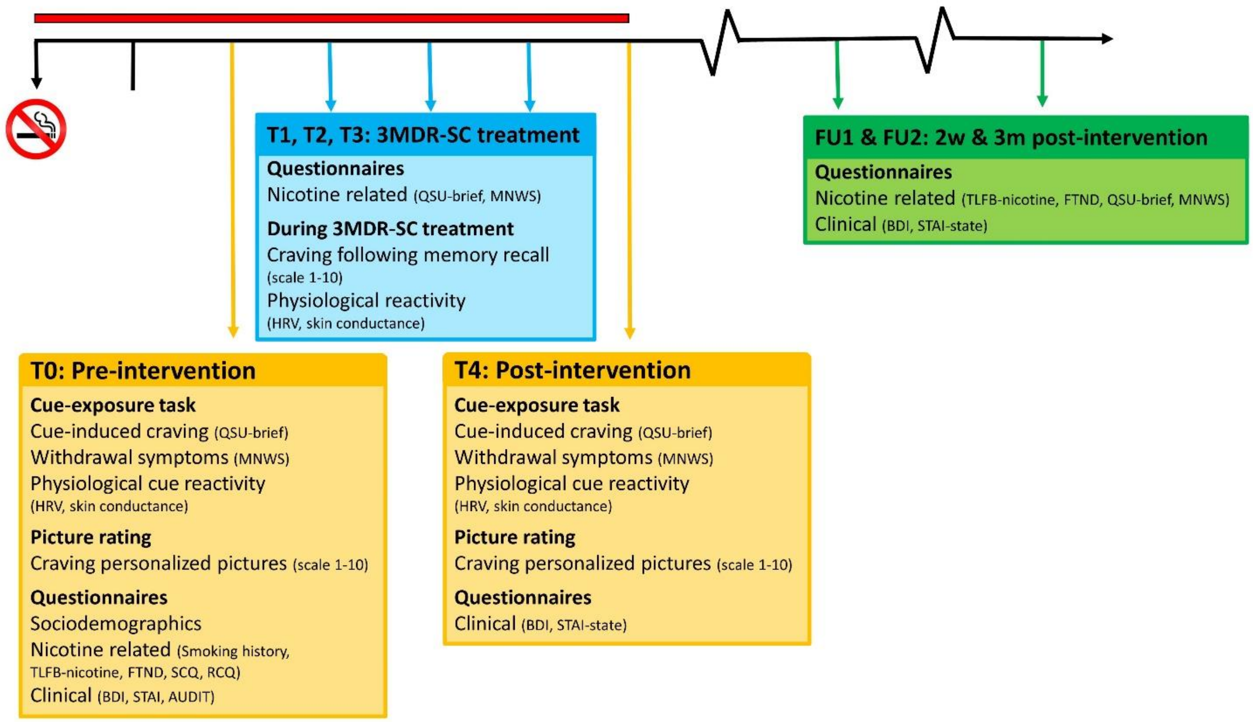

Figure S1. Overview of study procedures. Shown are the general study procedures pre-intervention at T0, during the three-day treatment at T1, T2 and T3, post-intervention at T4 and at 2-week (2w) and 3-month (3m) follow-up (FU1 and FU2). Forty-eight hours before the start of T0, participants were asked to stop smoking and to remain abstinent until T4.

3MDR-SC = smoking cessation motion-assisted memory desensitization and reprocessing, AUDIT = Alcohol Use Disorders Identification Test, BDI = Beck Depression Inventory, FTND = Fagerström Test for Nicotine Dependence, HRV = heart rate variability, MNWS = Minnesota Nicotine Withdrawal Scale, QSU-brief = brief Questionnaire on Smoking Urges, RCQ = Readiness to Change Questionnaire, SCQ = Smoking Consequence Questionnaire, STAI = State-Trait Anxiety Inventory, TLFB = Timeline Follow-Back method over last 14 days.

Smoking cue-exposure task blocks

The smoking cue-exposure paradigm was modified from a protocol by Kaag *et al.* (2018) and Germeroth *et al.* (2017) and programmed in E-prime (version 2). The 8 items from the Minnesota Nicotine Withdrawal (MNWS) followed the QSU-brief, to measure last 24-hour nicotine withdrawal symptoms (Hughes, 1992). First, an audio with guided relaxation was played for 75 seconds. The task then continued with two blocks of visual nicotine cues, with a 5-minute duration in total. Participants were instructed to look carefully at the images by trying to imagine themselves in the situations. In the first block, five video clips of 30 seconds were shown, depicting situations involving people smoking (adapted from Germeroth *et al.*, 2017). The second block consisted of a random presentation of 20 smoking-related pictures (adapted from Gorey *et al.*, 2023), 7.5 seconds each. Different pictures and video clips were shown to each participant per session. The task was followed by the handling of smoking paraphernalia, that was guided through four instructions shown on the computer screen for

30 seconds each (table 1). The lighter and package of cigarettes used during the handling block were taken from a closed white box on the table in front of the participant and were adjusted to the favorite brand of each participant.

Table S1. Instructions of handling smoking paraphernalia in the smoking cue-exposure task.

	**Instruction**
**1**	Open the box in front of you, put the lighter on the table in front of you and take the pack of cigarettes in your hands.
**2**	Take a cigarette out of the package and hold it between your fingers as you normally would when you start smoking.
**3**	Bring the cigarette to your face and give it a good sniff a few times.
**4**	Take the lighter and turn it on as you normally would if you were smoking, without actually lighting the cigarette.

HRV and skin conductance measurement details

HRV and *skin conductance* were measured by recording the participants electrocardiogram (ECG) and electrodermal activity (EDA) respectively, using the VU-AMS hardware and VU-DAMS software (version 5.4.13). During the 3MDR-SC intervention, the VU-AMS was attached to a belt worn by the participants to be able to record the EDA and ECG, and a VU-AMS remote device was used to manually place markers in the data at the moment current cigarette craving was assessed. After connecting the VU-AMS to an ECG V- lead electrode placed at the suprasternal notch, ECG V+ lead electrode between the bottom left third and fourth rib and ECG ground electrode between the right lower two ribs, ECG was acquired with a sampling frequency of 1000 Hz and -20 to 20 mV signal range. The EDA signal was measured on a 10 Hz sample frequency and 0-95 µS signal range utilizing a 16 bit A/D converter connected to an EDA electrode placed at the thenar eminence and an ECG electrode placed on the wrist bone. Disposable 55 mm diameter round ECG electrodes with an Ag/AgCl sensor and solid hydrogel (Kendall H98SG) and EDA electrodes of 27x36 mm with an Ag/AgCl sensor (Biopac EL507) were used. HRV was a derived parameter from the ECG signal and skin conductance from the EDA signal.

HRV and skin conductance processing

Physiological data recorded during the smoking cue-exposure task was processed using VU-DAMS software (version 5.4.13). R-peaks and suspicious inter-beat intervals in the raw ECG signal were automatically detected and marked by the software, as well as artefacts due to clipping or signal loss in the raw ECG and EDA signal. All data was visually inspected and manually corrected; artefacts and noisy data were removed, and R-peaks and beats were added, removed or adjusted if they were omitted or misplaced.

Thereafter, the data recorded during the smoking cue-exposure task was labeled by a relaxation, video presentation, picture presentation and handling phase, based on the corresponding blocks of the cue-exposure task. Each label was manually added between the two markers that were automatically placed by E-prime during data acquisition at the start and end of each task block.

The ECG and EDA data recorded during 3MDR-SC was manually labeled by a baseline, recall and task treatment phase for the three cues (cigarette, first and second picture). The baseline label was

placed during the 30 seconds in prior to the baseline memory recall marker. The recall labels were placed 20 seconds before and 10 seconds following memory recall of smoking cues before the task marker, a time window wherein the addiction memory was recalled. The task labels were placed 90 seconds prior to the memory recall after the distractor marker for a duration of 40 seconds, a time window wherein the distractor or non-distracting task was performed.

Per label, the RMSSD in ms, a time-based measure of HRV, and the mean SCL in μS, a tonic measure of EDA, was calculated by the VU-DAMS. Data of less than 10s per label was considered missing.

Additional questionnaires

At T0

Sociodemographic information, including age, sex assigned at birth (female or male), gender-identity (woman, man or none of both), country of origin, education (eight categories ranging from primary education to scientific education), work situation (5 categories, e.g. paid employment or unemployed), income (13 categories ranging from less than 500 to more than 10,000 euro/month gross) family situation (6 categories, e.g. married or single) and number of children (living at home) were assessed, as well as smoking history (e.g. onset age of regular smoking and number of quit attempts). The Smoking Consequence Questionnaire (SCQ) was used to assess smoking outcome expectancies (Myers *et al.*, 2003), and the valence of expectancies was provided for each scale of the SCQ, being negative consequences, positive reinforcement, negative reinforcement and appetite/weight control. To determine the stage of readiness to change, scores on the precontemplation, contemplation and action scales of the Readiness to Change Questionnaire (RCQ) were used (Rollnick *et al.*, 1992). Last 7-day depression symptoms were measured with the Beck Depression Inventory (BDI; Beck *et al.*, 1961), state and trait anxiety with the State-Trait Anxiety Inventory (STAI; Spielberger *et al.*, 1983) and alcohol use severity with the AUDIT.

At T1, T2 and T3

After the smokerlyzer breath test and before start of the 3MDR-SC treatment at T1, T2 and T3, the QSU-brief and MNWS were filled out with pen and paper by the participants to measure state-dependent craving and last 24-hour nicotine withdrawal symptom changes.

At T4

At the end of the last session, participants received online questionnaires that were filled out outside of the study location. These questionnaires included the BDI to measure last 7-day depression symptoms and the STAI-state to measure state-dependent anxiety.

At FU1 and FU2

State-dependent craving, last 7-day nicotine withdrawal symptoms, last 7-day depression symptoms and state anxiety were assessed using the QSU-brief, MNWS, BDI and STAI-state respectively.

At study completion

After the FU2 assessments of the last participant, all participants received an online survey asking if they had an idea what treatment they had received to assess treatment group awareness. The possible answers options were *1)* yes, the treatment that is expected to be most effective (interpret as active 3MDR-SC), *2)* yes, the treatment that may be less effective (interpret as control 3MDR-SC), or *3)* no, I do not know.

3MDR-SC treatment details

During the treatment, participants walked on the treadmill at a self-chosen and comfortable pace. A virtual landscape was projected on three 4x2.5 m screens surrounding the participant, creating a 180° panorama view, and passed by at the same speed as the treadmill. In this virtual landscape, participants walked through a tunnel with a visual stimulus at the end of it that enlarged to its maximum size when walking towards it. During the first cue exposure, this visual stimulus consisted of a grey plane as an in vivo cigarette instead of a visual stimulus was used to activate smoking memory. Specifically, participants were briefly taken off the treadmill and asked to light and take two puffs of the cigarette and to exhale the smoke in a fume hood. Thereafter, when the original walking speed on the treadmill was reached again, the participants were asked to recall the cigarette from a moment ago. A distractor or non-distractor task of 90 seconds then started while participants were asked to keep recalling the cigarette. After this task, participants walked out of the virtual tunnel and were asked to recall the cigarette from a moment ago again. This cue-exposure and distractor sequence was then repeated twice, but then the self-chosen pictures were used instead of the smoked cigarette to activate smoking memory. Participants were asked to explain why this picture was associated with their smoking behavior while walking through the tunnel towards the pictures, and were asked to recall the memory from the picture when the picture reached its maximum size.

The distractor task consisted of a red ball oscillating in a horizontal plane at 1Hz on the screen in the middle over the visual stimulus (grey plane or picture) and contained random changing numbers. Participants were instructed to follow the ball with their eyes while keeping their head still and simultaneously calling out the numbers on the ball. This was accompanied by binaural auditory stimulation (beeps) enabled by a surround sound system. The non-distractor task consisted of a stationary red ball that was presented in the middle of the screen over the presented visual stimulus. In this task, participants were instructed to keep their eyes on the ball.

Statistical analyses

Baseline differences between the active and control 3MDR-SC group were determined with t-tests (or non-parametric Mann-Whitney U test) and chi-square tests.

Main and secondary outcome analyses details

A paired sample t-test (or non-parametric Wilcoxon Signed-Ranks test) was performed for QSU-brief total sum score before and after cue exposure at T0 to check whether craving was induced by the nicotine cue-exposure task. The effect of active 3MDR-SC on cue-induced craving directly following treatment was tested with a rmANOVA on total (and reward- and relief-related) cue-induced craving

with session (T0 and T4) as a within-subject variable and group (active and control 3MDR-SC) as a between-subject factor.

A linear mixed model on HRV (in RMSSD) and skin conductance (in mean SCL) was performed with a random intercept and block (relax, video, picture and handling) as fixed effect at T0 to check whether physiological cue reactivity was present during the nicotine cue-exposure task. To investigate the effect of active 3MDR-SC on physiological cue-reactivity directly following treatment, a linear mixed model was performed on HRV and skin conductance with a random intercept and session (T0 and T4), block (relax, video, picture and handling), group and all interactions as fixed effects.

The effect of active 3MDR-SC on nicotine dependence severity and daily cigarette use at follow-up was tested with a rmANOVA on FTND sum scores and daily cigarette use, with time (T0, FU1 and FU2) as a within-subject variable and group as a between-subject factor.

Relapse rates were compared between the active and control 3MDR-SC group at FU1 and FU2 with chi-square tests.

Exploratory analyses details

To investigate the effect of active 3MDR-SC on craving following treatment for personalized pictures that were and were not selected during treatment, a rmANOVA on mean craving ratings (on a scale from 1 to 10) with session (T0 and T4) and picture type (selected and non-selected) as within-subject variables and group as a between-subject factor was performed.

A rmANOVA on craving (on a scale from 1 to 10) with session (T1, T2 and T3) and treatment phase (at baseline, following memory recall before and after the task for three cues) as within-subject variables and group as a between-subject factor was done to see the effect of active 3MDR-SC on craving following memory recall during the three-day treatment.

The effect of active 3MDR-SC on craving and withdrawal symptoms during treatment was investigated with a rmANOVA on QSU-brief and MNWS sum score, with time (T0, T1, T2, T3, T4, FU1 and FU2) as a within-subject variable and group (active and control 3MDR-SC) as a between-subject factor.

A rmANOVA on BDI and STAI-state sum score with time (T0, T4, FU1 and FU2) as a within-subject variable and group as a between-subject factor was performed, to determine whether there was an effect of active 3MDR-SC on depression symptoms and state-dependent anxiety following treatment and follow-up.

To assess the relationship between depression and withdrawal symptoms and between state-dependent anxiety and withdrawal symptoms, a one-tailed Pearson correlation test was performed with either the BDI or STAI-state sum scores and MNWS sum scores assessed at multiple time points (T0, T4, FU1 and FU2) for both groups (active and control 3MDR-SC) combined. A Kendall's tau-b correlation was done in case the assumption of normality and/or homoscedasticity was violated.

A linear mixed model on HRV (RMSSD) and skin conductance (mean SCL) with a random intercept and session (T1, T2 and T3), treatment phase (baseline, during memory recall before task and during task for three cues), group and all interactions as fixed effects was performed to investigate the effect of active 3MDR-SC on physiological cue- and task-reactivity during treatment was investigated.

SPSS (version 28) was used to perform the data-analyses and a $p < 0.05$ was considered significant. A $\eta_{(p)}^2 < 0.06$ was considered a small effect, a $0.06 \leq \eta_{(p)}^2 < 0.14$ was considered a medium effect and $\eta_{(p)}^2 \geq 0.14$ a large effect. A Greenhouse-Geisser or Huynh-Feldt correction was applied in case the

violation of sphericity was violated in all rmANOVAs. Imputation was applied when a single item was missing in a multiple-item questionnaire. Figures were created with GraphPad Prism (version 9.3.1).

Supplementary results

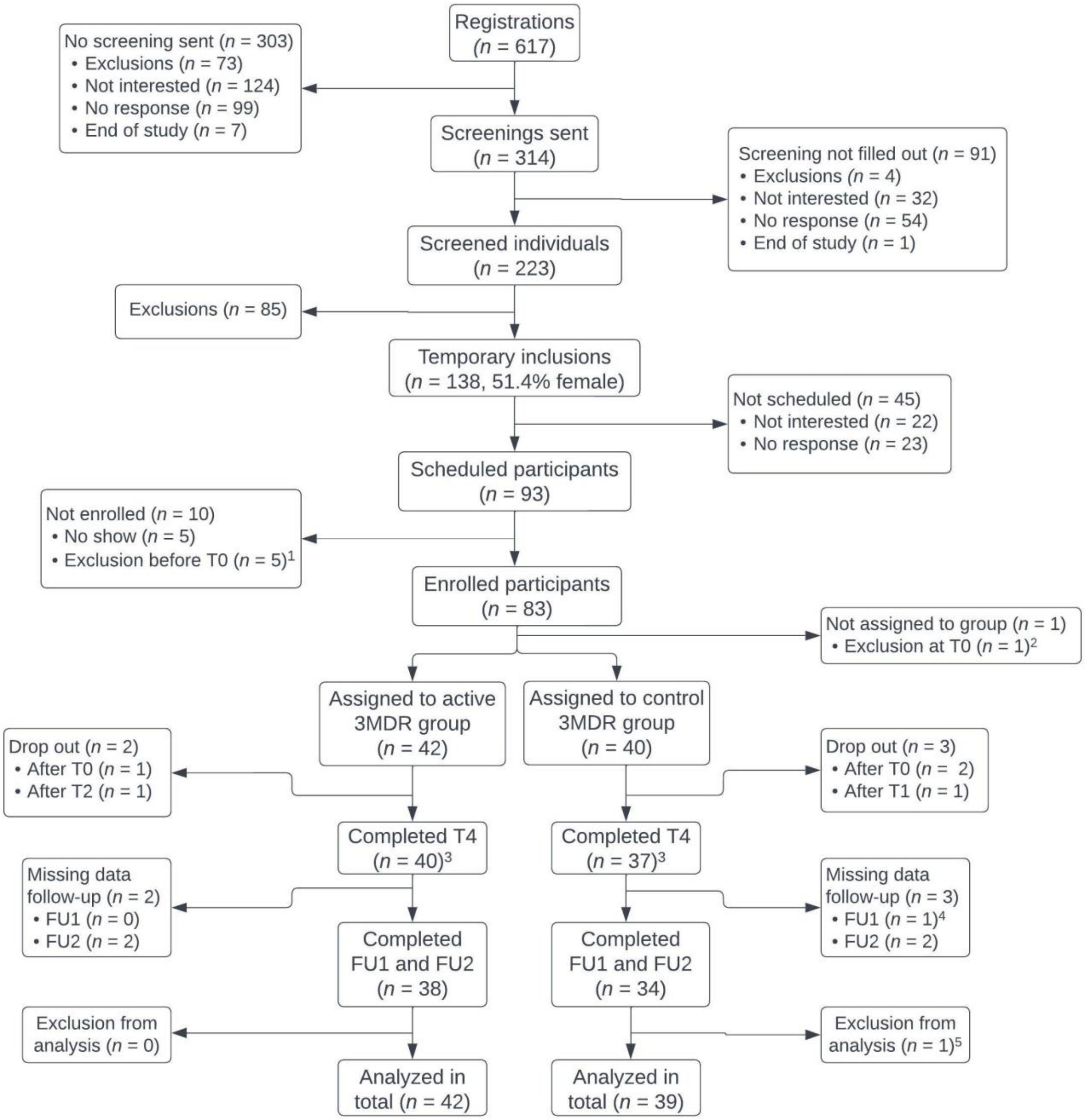

Figure S2. Flow-chart of individuals involved in study. Shown are the number of registered individuals and those that eventually did and did not get screened, included, scheduled, enrolled, randomly assigned, completed the treatment, completed the follow-up phases and got analyzed.

Note: 1) Exclusion before T0 (*n* = 5) was due to not being able to remain abstinent from smoking 48 hours before T0 as self-indicated by the participants. *2)* Exclusion at T0 (*n* = 1) was due to exhaled carbon monoxide levels exceeding 10 parts per million. *3)* Of the T4 completers, a participant from the control group (*n* = 1) did not perform the cue-exposure task at T4 and a participant from the active group (*n* = 1) did not complete the questionnaires at T4 (also not at FU2; therefore considered lost to follow-up). *4)* Participant from control group with missing data at FU1 (*n* = 1) did not have missing data at FU2. *5)* Exclusion from analysis (*n* = 1) was due to current use of psychotropic medication (an exclusion criterion) declared by the participant at T2. Analyses of the main and secondary outcomes revealed the same significant results when this participant was not excluded from the dataset.

Table S2. Baseline sociodemographic, nicotine-related, clinical and study-related characteristics from the active 3MDR-SC and control 3MDR-SC group.

		Active 3MDR-SC (n = 42)	*Control 3MDR-SC (n = 39)*	*Test statistics*
Sociodemographics				
Age		43.5, 14.0[a]	42.0, 13.0[a]	U = 797.500, p = .839
Sex assigned at birth[1]	Male	45.2% (n = 19)	53.8% (n = 21)	
	Female	54.8% (n = 23)	46.2% (n = 18)	χ^2_1 = .599, p = .439
Country of origin	Netherlands	97.6% (n = 41)	87.2% (n = 34)	χ^2_5 = 6.551, p = .256
	Other countries	2.4% (n = 1)	12.8% (n = 5)	
Education	Primary/secondary education	47.6% (n = 20)	41.0% (n = 16)	U = 800.500, p = .855
	Higher education	52.4% (n = 22)	59.0% (n = 23)	
Work situation	Paid employment	59.5% (n = 25)	71.8% (n = 28)	χ^2_5 = 3.868, p = .569
	Other (freelancer, unemployed, volunteer work or in education)	40.5% (n = 17)	28.2% (n = 11)	
Income	≤€3000-4000 per month	54.8% (n = 23)	43.6% (n = 17)	U = 673.0, p = .163
	>€3000-4000 per month	45.2% (n = 19)	56.4% (n = 22)	
Family situation	Married or living together	52.4% (n = 22)	56.4% (n = 22)	χ^2_5 = 6.852, p = .232
	Other (unmarried, divorced, single or widowed)	47.6% (n = 20)	43.6% (n = 17)	
Number of children		1, 2[a]	1, 2[a]	U = 802.500, p = .868
Number of children living at home		1, 1[a]	0, 1[a]	U = 773.500, p = .637
Nicotine related				
Daily current cigarette use		14.7, 6.1[b]	14.4, 5.9[b]	t_{79} = .174, p = .862
Years since regular cigarette use		24.9, 9.0[b]	24.4, 7.2[b]	t_{79} = .246, p = .806
Daily cigarette use at onset		5.0, 5.0[a]	8.0, 5.0[a]	U = 694.000, p = .229
Years since onset cigarette use		27.5, 16.0[a]	27.0, 11.0[a]	U = 791.500, p = .795
Weekly cigarette expenses		36.4, 18.3[b]	38.3, 20.8[b]	t_{79} = -.426, p = .671
Number of quit attempts		4, 3[a]	4, 6[a]	U = 748.500, p = .503
Number of quit attempt >24h		3, 3[a]	3, 4[a]	U = 797.500, p = .837
Days of quit attempt last 10 years[2]		55, 245[a]	90, 215[a]	U = 668.000, p = .210
FTND		5.0, 3.0[a]	5.0, 3.0[a]	U = 761.000, p = .580
SCQ	Negative consequences	35.0, 3.0[a]	36.0, 1.0[a]	U = 645.500, p = .079
	Positive reinforcement	28.0, 11.0[a]	27.0, 17.0[a]	U = 802.500, p = .876
	Negative reinforcement	39.5, 15.0[a]	41.0, 25.0[a]	U = 796.000, p = .828
	Appetite-weight control	21.5, 22.0[a]	25.0, 22.0[a]	U = 741.500, p = .463
RCQ[3]	Action	38.1% (n = 16)	30.8% (n = 12)	χ^2_1 = .480, p = .488
	Contemplation	61.9% (n = 26)	69.2% (n = 27)	

QSU-brief		27.0, 19.0[a]	33.0, 20.0[a]	U = 703.000, p = .273
MNWS		16.3, 6.2[b]	17.1, 5.9[b]	t_{79} = -.591, p = .556
Clinical				
BDI		6.5, 7.0[a]	6.0, 9.0[a]	U = 766.000, p = .615
STAI-trait		41.1, 8.3[b]	42.4, 8.7[b]	t_{79} = -.659, p = .512
STAI-state		43.7, 9.8[b]	42.4, 8.5[b]	t_{79} = .625, p = .534
AUDIT		7.0, 5.0[a]	6.0, 7.0[a]	U = 743.000, p = .471
Study related				
Group awareness[4]	Active 3MDR-SC	11.4% (n = 4)	16.0% (n = 4)	χ^2_2 = 3.093, p = .213
	Control 3MDR-SC	25.7% (n = 9)	8.0% (n = 2)	
	Do not know	62.9% (n = 22)	76.0% (n = 19)	
Missing data FU1		4.8% (n = 2)	10.3% (n = 4)	χ^2_1 = .890, p = .345
Missing data FU2		9.5% (n = 4)	12.8% (n = 5)	χ^2_1 = .223, p = .637
Days late of filling out FU1		0.0, 2.0[a]	0.0, 1.0[a]	U = 687.000, p = .873
Days late of filling out FU2		1.0, 2.0[a]	0.0, 1.0[a]	U = 519.000, p = .117
Days since last cigarette		2.0, 1.0[a]	2.0, 1.0[a]	U = 816.000, p = .975

Shown per group, with number of participants (n), are the [a]mean and standard deviation (SD) of parametric values, the [b]median and interquartile range (IQR) of non-parametric values and percentages of categorical values, as well as the corresponding test statistics of the t-test (t), Mann-Whitney U test (U) or chi-square test (χ^2) assessing group differences for these values.

Note: 1) Although missing data of gender-identity was present for participants in the active (n = 19) and control 3MDR-SC (n = 16) group, all questioned participants had a gender-identity that matched their sex assigned at birth. *2)* Missing data of days of quit attempts last 10 years for a participant in the control 3MDR-SC group (n = 1). *3)* No participants were allocated to the RCQ pre-contemplation stage. *4)* Missing data of group awareness for participants in the active (n = 7) and control 3MDR-SC (n = 14) group.

Table S3. Results of the rmANOVA on the main study outcome: cue-induced craving at T0 and T4.

Source	*SS*	*df*	*MS*	*F*	*p*	η_p^2
Between-subjects effects on total cue-induced craving						
**Intercept**	1491.669	1	1491.669	41.931	**<.001****	.365
**Group**	32.069	1	32.069	.901	.346	.012
**Error**	2596.918	73	35.574			
Within-subjects effects on total cue-induced craving						
**Session * group**	59.002	1	59.002	1.171	.283	.016
**Session**	770.469	1	770.469	15.293	**< .001****	.173
**Error**	3677.771	73	50.380			
Between-subjects effects on reward-related cue-induced craving						
**Intercept**	527.886	1	527.886	38.254	**<.001****	.344
**Group**	7.459	1	7.459	.541	.465	.007
**Error**	1007.361	73	13.799			
Within-subject effects on reward-related cue-induced craving						
**Session * group**	41.440	1	41.440	2.430	.123	.032
**Session**	180.107	1	180.107	10.561	**.002***	.126
**Error**	1244.893	73	17.053			
Between-subjects effects on relief-related cue-induced craving						
**Intercept**	244.809	1	244.809	30.676	**<.001****	.296
**Group**	8.595	1	8.595	1.077	.303	.015
**Error**	582.571	73	7.980			
Between-subject effects on relief-related cue-induced craving						
**Session * group**	1.547	1	1.547	.125	.724	.002
**Session**	205.547	1	205.547	16.670	**< .001****	.186
**Error**	900.093	73	12.330			

Shown is the source, sum of squares (SS), degrees of freedom (df), mean square (MS), F-value (*F*), p-value (*p*), and partial eta squared (η_p^2) of within-subjects effects. *, $p < 0.05$. **, $p < 0.001$.

Table S4. Results of the linear mixed models on the secondary study outcome: HRV and skin conductance cue-reactivity at T0, and at T0 and T4.

Source	df_{num}	df_{den}	*F*	*p*	η^2
Fixed effects on HRV at T0					
**Block**	3	210.203	40.857	**<.001****	0.368
**Intercept**	1	68.070	436.549	**<.001****	
Fixed effects on skin conductance at T0					
**Block**	3	233.057	47.735	**<.001****	0.381
**Intercept**	1	78.004	140.325	**<.001****	

Comparison	*M difference*	*SE*	*df*	*P*	*95% CI*
Pairwise comparisons on HRV at T0					
**R-V**	-11.592	1.330	210.317	**<.001****	-14.801, -8.383
**R-P**	-13.694	1.336	210.477	**<.001****	-16.919, -10.469
**R-H**	-8.760	1.342	210.552	**<.001****	-11.999, -5.520
Pairwise comparisons on skin conductance at T0					
**R-V**	.000	.205	233.052	1.000	-.494, .495
**R-P**	-.311	.205	233.052	.394	-.805, .184
**R-H**	1.892	.206	233.061	**<.001****	1.395, 2.388

Source	*df_{num}*	*df_{den}*	*F*	*p*	*η^2*
Fixed effects on HRV at T0 and T4					
**Session * block * group**	3	483.892	.070	.976	< .001
**Session * group**	1	495.766	.009	.925	< .001
**Block * group**	3	483.885	.642	.588	.003
**Session * block**	3	483.892	.301	.824	.002
**Group**	1	78.700	.015	.903	< .001
**Session**	1	495.766	9.604	**.002***	.019
**Block**	3	483.885	38.253	**< .001****	.192
**Intercept**	1	78.700	387.918	**< .001****	
Fixed effects on skin conductance at T0 and T4					
**Session * block * group**	3	508.139	.045	.987	< .001
**Session * group**	1	512.897	.446	.505	< .001
**Block * group**	3	508.139	.425	.736	.003
**Session * block**	3	508.139	.176	.913	< .001
**Group**	1	79.128	1.949	.167	.024
**Session**	1	512.897	.103	.749	< .001
**Block**	3	508.139	18.171	**< .001****	.096
**Intercept**	1	79.128	218.921	**< .001****	

Comparison	*M difference*	*SE*	*df*	*P*	*95% CI*
Pairwise comparisons on HRV at T0 and T4					
**T0-T4**	3.166	1.022	495.766	**.002***	1.159, 5.173
**R-V**	11.659	1.368	483.895	**< .001****	8.034, 15.284
**R-P**	13.314	1.364	483.864	**< .001****	9.701, 16.927
**R-H**	9.636	1.369	483.915	**< .001****	6.010, 13.262
Pairwise comparisons on skin conductance at T0 and T4					
**R-V**	-.076	.372	508.132	1.000	-1.060, .909
**R-P**	.236	.372	508.132	1.000	-.748, 1.220
**R-H**	-2.177	.372	508.147	**< .001****	-3.162, -1.191

Shown is the source, degrees of freedom of the numerator (df_{num}) and denominator (df_{den}), F-value (*F*), p-value (*p*), and eta squared (η_p^2) of fixed effects, and the mean (M) difference, standard error (SE), degrees of freedom (df), p-value (*p*), and 95% confidence interval (CI) of pairwise comparisons. *, $p < 0.05$. **, $p < 0.001$. *R = relaxation, V = video, P = picture, H = handling.*

Table S5. Results of the rmANOVA on the secondary outcome: FTND sum score and daily cigarette use at T0, FU1 and FU2.

Source	*SS*	*df*	*MS*	*F*	*p*	*η_p^2*
Between-subject effects on FTND sum score						
**Intercept**	951.130	1	951.130	298.348	**<.001****	.812
**Group**	1.903	1	1.903	.597	.442	.009
**Error**	219.971	69	3.188			
Within-subject effects on FTND sum score						
**Time * group**	2.949	1.865	1.582	.960	.380	.014
**Time**	75.099	1.865	40.276	24.450	**< .001****	.262
**Error**	211.934	128.659	1.647			
Simple within-subject contrasts on FTND sum score						
**Time: FU1 vs. T0**	121.721	1	121.721	33.366	**< .001****	.326
**Error**	251.716	69	3.648			
**Time: FU2 vs. T0**	102.777	1	102.777	50.619	**< .001****	.423
**Error**	140.096	69	2.030			

Between-subject effects on daily cigarette use						
**Intercept**	4626.816	1	4626.816	208.699	**<.001****	.752
**Group**	10.373	1	10.373	.468	.496	.007
**Error**	1529.714	69	22.170			
Within-subjects effects on daily cigarette use						
**Time * group**	.785	2	.393	.019	.981	.000
**Time**	4717.684	2	2358.842	115.397	**< .001****	.626
**Error**	2820.872	138	20.441			
Simple within-subject contrasts on daily cigarette use						
**Time: FU1 vs. T0**	9153.820	1	9153.820	207.875	**< .001****	.751
**Error**	3038.431	69	44.035			
**Time: FU2 vs. T0**	3889.915	1	3889.915	90.888	**< .001****	.568
**Error**	2953.127	69	42.799			

Shown is the source, sum of squares (SS), degrees of freedom (df), mean square (MS), F-value (*F*), p-value (*p*), and partial eta squared (η_p^2) of within-subjects effects and contrasts. *, $p < 0.05$. **, $p < 0.001$.

Table S6. Results of the rmANOVA on craving for personalized pictures at T0 and T4.

Source	***SS***	***df***	***MS***	***F***	***p***	η_p^2
Between-subject effects						
Intercept	2082.730	1	2082.730	1282.341	**<.001****	.945
Group	.721	1	.721	.444	.507	.006
Error	120.188	74	1.624			
Within-subject effects						
**Session * picture type * group**	.036	1	.036	.120	.730	.002
**Session * picture type**	18.943	1	18.943	63.070	**<.001****	.460
**Error**	22.226	74	.300	22.226	74	.300
**Session * group**	5.238	1	5.238	1.269	.264	.017
**Error**	305.543	74	4.129			
**Picture type * group**	1.970	1	1.970	2.338	.131	.031
**Error**	62.343	74	.842			
Within-subject effects per picture type						
**Selected: session**	516.404	1	516.404	219.436	**<.001****	.745
**Error**	176.499	75	2.353			
**Non-selected: session**	274.730	1	274.730	131.623	**<.001****	.637
**Error**	156.544	75	2.087			
Within-subject effects per session						
**T0: Picture type**	176.552	1	176.552	266.499	**<.001****	.780
**Error**	49.686	75	.662			
**T4: Picture type**	50.947	1	50.947	103.586	**<.001****	.580
**Error**	36.888	75	.492			

Shown is the source, sum of squares (SS), degrees of freedom (df), mean square (MS), F-value (*F*), p-value (*p*), and partial eta squared (η_p^2) of within-subjects effects. *, $p < 0.05$. **, $p < 0.001$.

Table S7. Results of the rmANOVA on craving following memory recall of cigarette-related cues during at T1, T2 and T3.

Source	*SS*	*df*	*MS*	*F*	*p*	η_p^2
Between-subject effects						
**Intercept**	1444.900	1	1444.900	451.087	**<.001****	.859
**Group**	.430	1	.430	.134	.715	.002
**Error**	237.033	74	3.203			
Within-subject effects						
**Session * treatment phase * group**	39.164	7.739	5.061	2.163	**.030***	.028
**Error**	1340.029	572.671	2.340			
Within-subject effects per treatment phase…						
**B: session * group**	9.640	1.899	5.076	1.904	.155	.025
**Error**	374.737	140.549	2.666			
**C: session * group**	12.248	2.000	6.124	1.859	.159	.025
**Error**	487.447	148.000	487.447			
**T(C): session * group**	12.913	1.703	7.580	3.398	**.044***	.044
**Error**	281.228	126.055	2.231			
Repeated within-subject contrasts for T(C) treatment phase						
**Session * group: T1-T2**	21.783	1	21.783	6.652	**.012***	.082
**Error**	242.322	74	3.275			
**Session * group: T2-T3**	.351	1	.351	.134	.715	.002
**Error**	193.281	74	2.612			
Continuation of within-subject effects per treatment phase						
**P1: session * group**	1.651	1.870	.883	.253	.762	.003
**Error**	483.831	138.412	3.496			
**T(P1): session * group**	5.957	1.825	3.264	1.487	.230	.020
**Error**	296.420	135.066	2.195			
**P2: session * group**	3.306	2	1.653	.731	.483	.010
**Error**	334.826	148	2.262			
**T(P2): session * group**	8.835	2	4.417	3.164	**.045***	.041
**Error**	206.654	148	1.396			
Repeated within-subject contrasts for T(P2) treatment phase						
**Session * group: T1-T2**	10.976	1	10.976	3.969	**.050***	.051
**Error**	204.656	74	2.766			
**Session * group: T2-T3**	.341	1	.341	.128	.722	.002
**Error**	197.148	74	2.664			

Shown is the source, sum of squares (SS), degrees of freedom (df), mean square (MS), F-value (*F*), p-value (*p*), and partial eta squared (η_p^2) of within-subjects effects and contrasts. *, $p <$ (and ≤) 0.05. **, $p < 0.001$. *B = baseline, C = puffed cigarette, T(C) = puffed cigarette after the (non-)distractor task, P1 = first personalized picture, T(P1) = first personalized picture after the (non-)distractor task, P2 = second personalized picture, T(P2) = second personalized picture after the (non-)distractor task.*

Results from exploratory analyses

Craving (QSU-brief) and withdrawal scores

There was no significant interaction between time (T0, T1, T2, T3, T4, FU1 and FU2) * group (active and control) for the QSU-brief sum score and MNWS sum score, but both sum scores showed a significant large main effect of time, independent of group. Polynomial contrasts showed that this main effect of time on the QSU-brief sum score was primarily quadratic and on the MNWS sum score was primarily linear. Moreover, simple contrasts for the QSU-brief and MNWS sum score revealed a

significant reduction from T0 to all other study session and follow-up time points. See figure S3 and table S8 for all statistics.

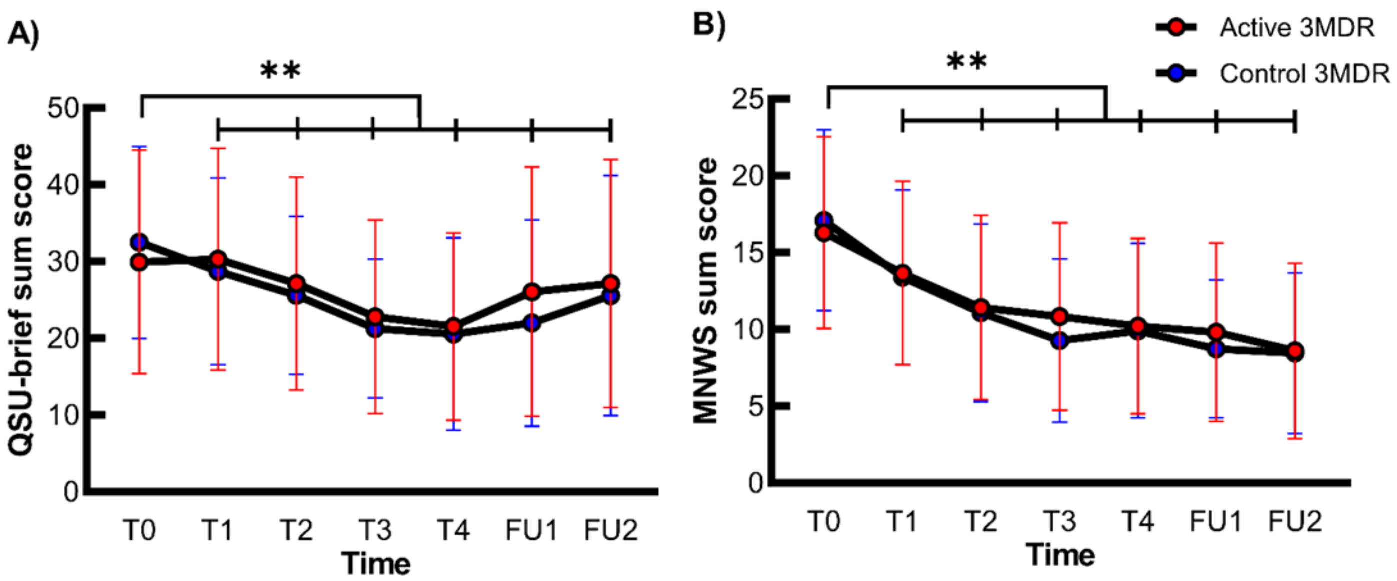

Figure S3. The effect of active 3MDR-SC on craving and withdrawal symptoms during and after treatment at follow-up. Shown is the mean (+/- SD) QSU-brief sum score ***(A)*** and MNWS sum score ***(B)*** at T0 up to T4 and at FU1 and FU2 in the active 3MDR-SC (red) and control 3MDR-SC (blue) group (n = 69 for the QSU-brief and n = 70 for the MNWS sum score). **, main effect of time of $p < .001$.

Note: In addition to missing data mentioned in figure S2 (see third note), missing data was also present for the QSU-brief sum score at T3 for a participant in control group (n = 1).

Table S8. Results of the rmANOVA on QSU-brief and MNWS sum score during T0-T4 and at FU1 and FU2.

Source	*SS*	*df*	*MS*	*F*	*p*	η_p^2
Between-subject effects on QSU-brief sum score						
**Intercept**	43933.485	1	43933.485	423.229	**<.001****	.863
**Group**	92.837	1	92.837	.894	.348	.013
**Error**	6954.963	67	103.805			
Within-subject effects on QSU-brief sum score						
**Time * group**	273.767	3.431	79.783	.569	.659	.008
**Time**	6067.324	3.431	1768.176	12.608	**< .001****	.158
**Error**	32242.850	229.904	140.245			
Polynomial within-subject contrasts on QSU-brief sum score						
**Time: linear**	2609.867	1	2609.867	12.534	**< .001****	.158
**Error**	13951.413	67	208.230			
**Time: quadratic**	2715.427	1	2715.427	28.875	**< .001****	.301
**Error**	6300.725	67	94.041			
**Time: cubic**	439.357	1	439.357	6.803	**.011***	.092
**Error**	4327.358	67	64.587			
**Time: order 4**	202.061	1	202.061	4.543	**.037***	.064
**Error**	2979.903	67	44.476			
**Time: order 5**	100.384	1	100.384	3.376	.071	.048

**Error**	1992.100	67	29.733			
Simple within-subject contrasts on QSU-brief sum score						
**Time: T1 vs. T0**	227.691	1	227.691	4.569	**.036***	.064
**Error**	3339.178	67	49.838			
**Time: T2 vs. T0**	1664.331	1	1664.331	14.343	**< .001****	.176
**Error**	7774.278	67	116.034			
**Time: T3 vs. T0**	5842.515	1	5842.515	52.077	**< .001****	.437
**Error**	7516.703	67	112.190			
**Time: T4 vs. T0**	7770.692	1	7770.692	49.222	**< .001****	.424
**Error**	10577.250	67	157.869			
**Time: FU1 vs. T0**	3853.488	1	3853.488	18.419	**< .001****	.216
**Error**	14017.063	67	209.210			
**Time: FU2 vs. T0**	1848.910	1	1848.910	6.071	**.016***	.083
**Error**	20404.424	67	304.544			
Between-subject effects on MNWS sum score						
**Intercept**	8797.069	1	8797.069	485.899	**<.001****	.877
**Group**	.548	1	.548	.030	.862	.000
**Error**	1231.123	68	18.105			
Within-subjects effects on MNWS sum score						
**Time * group**	38.128	3.953	9.646	.420	.792	.006
**Time**	3649.485	3.953	923.311	40.159	**< .001****	.371
**Error**	6179.595	268.777	22.992			
Polynomial within-subject contrasts on MNWS sum score						
**Time: linear**	3165.180	1	3165.180	90.815	**< .001****	.572
**Error**	2369.995	68	34.853			
**Time: quadratic**	408.742	1	408.742	23.188	**< .001****	.254
**Error**	1198.654	68	17.627			
**Time: cubic**	63.336	1	63.336	5.496	**.022***	.075
**Error**	783.633	68	11.524			
**Time: order 4**	2.604	1	2.604	.230	.633	.003
**Error**	771.029	68	11.339			
**Time: order 5**	.918	1	.918	.115	.735	.002
**Error**	542.341	68	7.976			
Simple within-subject contrasts on MNWS sum score						
**Time: T1 vs. T0**	729.863	1	729.863	33.141	**< .001****	.328
**Error**	1497.541	68	22.023			
**Time: T2 vs. T0**	1954.399	1	1954.399	74.864	**< .001****	.524
**Error**	1775.211	68	26.106			
**Time: T3 vs. T0**	3285.857	1	3285.857	114.877	**< .001****	.628
**Error**	1945.030	68	28.603			
**Time: T3 vs. T0**	3355.825	1	3355.825	102.127	**< .001****	.600
**Error**	2234.438	68	32.859			
**Time: FU1 vs. T0**	4081.004	1	4081.004	92.055	**< .001****	.575
**Error**	3014.606	68	44.332			
**Time: FU2 vs. T0**	4911.446	1	4911.446	109.783	**< .001****	.618
**Error**	3042.172	68	44.738			

Shown is the source, sum of squares (SS), degrees of freedom (df), mean square (MS), F-value (*F*), p-value (*p*), and partial eta squared (η_p^2) of within-subjects effects and contrasts. *, $p < 0.05$. **, $p < 0.001$.

<u>*Clinical symptoms*</u>

There was for BDI and STAI-state sum score no significant time (T0, T4, FU1 and FU2) * group (active and control) interaction effect. A significant large main effect of time was found, with simple contrasts

revealing a significant reduction of BDI and STAI-state sum score from T0 to T4, from T0 to FU1 and from T0 to FU2, independent of treatment group. See figure S4 and table S9 for all statistics.

There was a strong significant positive correlation between BDI and MNWS sum score ($\tau_b = .411$, $p < .001$), independent of time and treatment group. A strong significant positive correlation was also apparent between STAI-state and MNWS sum score ($\tau_b = .481$, $p < .001$).

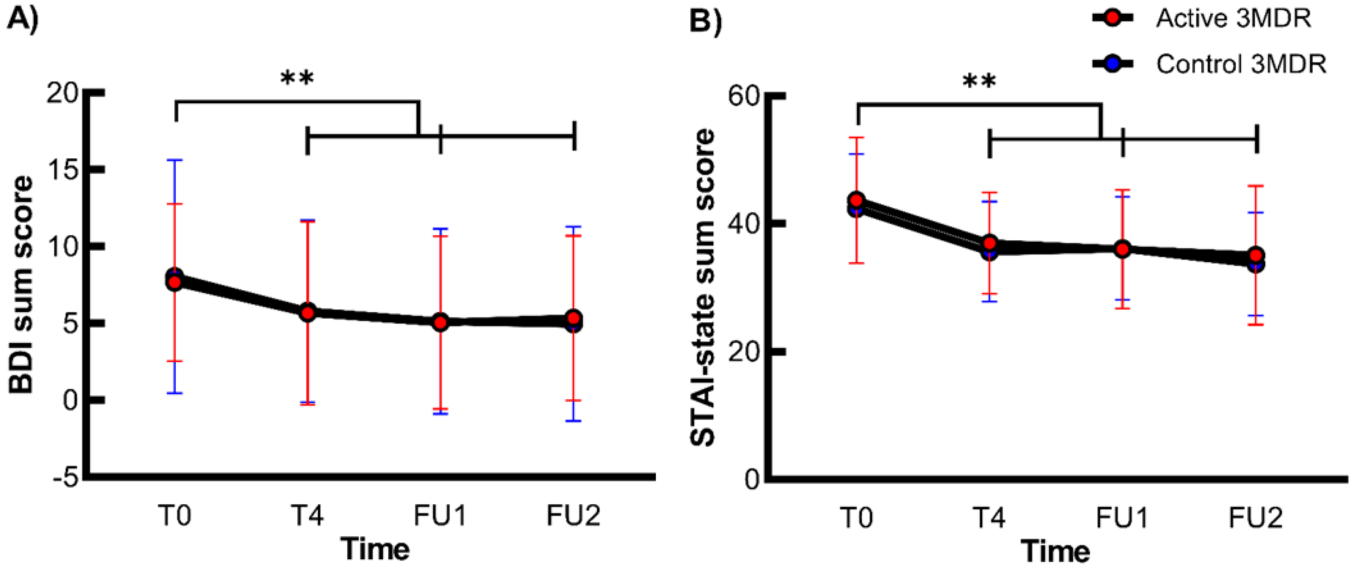

Figure S4. Depression symptoms and state-dependent anxiety before, directly following treatment and at follow-up. Shown is the mean (+/- SD) BDI sum score ***(A)*** and STAI-state sum score ***(B)*** at session T0, T4, FU1 and FU2 in the active 3MDR-SC (red) and control 3MDR-SC (blue) group (n = 71). **, main effect of time of $p < .001$.

Table S9. Results of the rmANOVA on BDI and STAI-state sum score at T0, T4, FU1 and FU2, directly following treatment and at follow-up.

Source	***SS***	***df***	***MS***	***F***	***p***	η_p^2
Between-subject effects on BDI sum score						
**Intercept**	2259.572	1	2259.572	94.173	**<.001****	.577
**Group**	2.639	1	2.639	.110	.741	.002
**Error**	1655.583	69	23.994			
Within-subjects effects on BDI sum score						
**Time * group**	17.132	2.878	5.953	.599	.610	.009
**Time**	337.963	2.878	117.428	11.814	**< .001****	.146
**Error**	1973.953	198.585	9.940			
Simple within-subjects contrasts on BDI sum score						
**Time: T4 vs. T0**	287.685	1	287.685	13.848	**< .001****	.167
**Error**	1433.414	69	20.774			
**Time: FU1 vs. T0**	483.044	1	483.044	26.444	**< .001****	.277
**Error**	1260.392	69	18.267			
**Time: FU2 vs. T0**	520.881	1	520.881	22.582	**< .001****	.247
**Error**	1591.598	69	23.067			
Between-subject effects on STAI-state sum score						
**Intercept**	951.130	1	951.130	298.348	**<.001****	.812

**Group**	1.903	1	1.903	.597	.442	.009
**Error**	219.971	69	3.188			
Within-subject effects on STAI-state sum score						
**Time * group**	39.095	2.772	14.106	.284	.821	.004
**Time**	3659.180	2.772	1320.280	26.592	**<.001****	.278
**Error**	9494.820	191.235	49.650			
Simple within-subjects contrasts on STAI-state sum score						
**Time: T4 vs. T0**	3840.713	1	3840.713	46.363	**< .001****	.402
**Error**	5716.019	69	82.841			
**Time: FU1 vs. T0**	4371.739	1	4371.739	48.188	**< .001****	.411
**Error**	6259.838	69	90.722			
**Time: FU2 vs. T0**	6028.666	1	6028.666	44.821	**< .001****	.394
**Error**	9280.884	69	134.506			

Shown is the source, sum of squares (SS), degrees of freedom (df), mean square (MS), F-value (*F*), p-value (*p*), and partial eta squared (η_p^2) of within-subjects effects and contrasts. *, $p < 0.05$. **, $p < 0.001$.

Physiological reactivity during 3MDR-SC

There was no significant three-way interaction between session (T1, T2 and T3) * treatment phase (baseline, recall and task for three smoking cues) * group. There was, however, a significant small interaction between session * group for HRV and SC, and pairwise comparisons for the groups separately revealed a significant main effect of session in the control group, with higher HRV and skin conductance at T1 as compared to the other sessions, while no significant main effect of session was present in the active 3MDR-SC group. Thus, lower HRV and skin conductance was found in the active as compared to the control 3MDR-SC group only at T1, independent of treatment phase. For all statistics, see figure S5 and table S10.

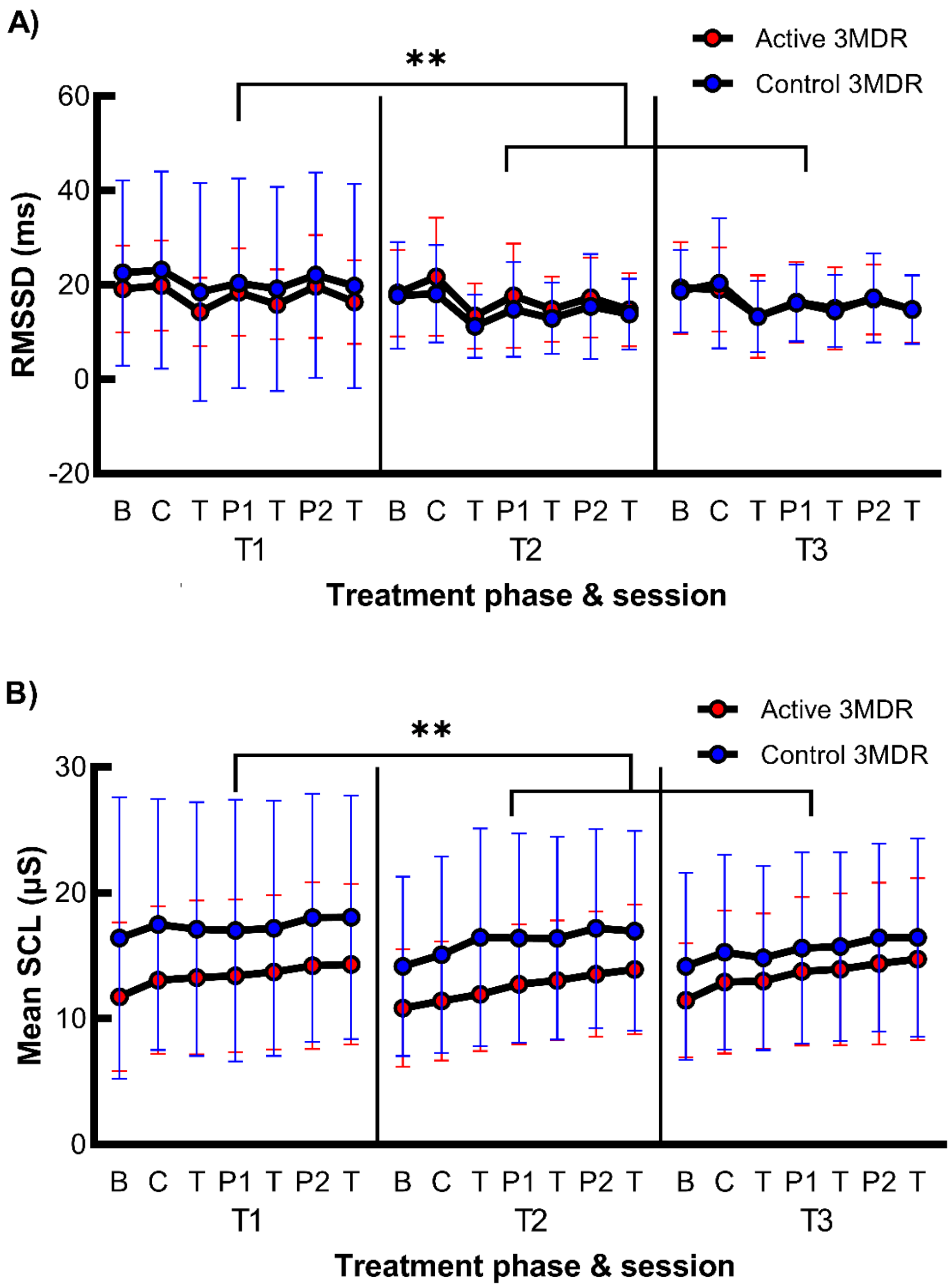

Figure S5. The effect of active 3MDR-SC on HRV and skin conductance reactivity during the three-day treatment. Shown is the mean (+/- SD) RMSSD ***(A)*** and mean SCL ***(B)*** at baseline (B), during recall of a puffed cigarette (C), first personalized picture (P1) or second personalized picture (P2) before the (non-)distractor task, and during each (non-)distractor task (T) in the active 3MDR-SC group (red) and control 3MDR-SC group (blue) at session T1, T2 and T3 (n = 79). **, session * condition interaction effect of $p < .001$. *B = baseline, C = puffed cigarette, T = (non-)distractor task, P1 = first personalized picture, P2 = second personalized picture.*

Note: Of the HRV data, 7.6% of the data points were missing (n = 125), and of the skin conductance data, 4.7% of the data points were missing (n = 77).

Table S10. Results of the linear mixed models on HRV and skin conductance reactivity at T1, T2 and T3.

Source	df_{num}	df_{den}	*F*	*p*	η^2
Fixed effects on HRV					
**Session * phase * group**	12	1395.330	.153	1.000	.001
**Session * group**	2	1405.032	7.309	**< .001****	.010
**Phase * group**	6	1395.417	.082	.998	< .001
**Session * phase**	12	1395.330	.225	.997	.002
**Group**	1	76.061	.191	.663	.003
**Session**	2	1405.032	15.854	**< .001****	.022
**Phase**	6	1395.417	12.705	**< .001****	.052
**Intercept**	1	76.061	365.749	**< .001****	
Fixed effects on HRV for the active 3MDR-SC group					
**Session**	2	728.758	2.798	.062	.008
Fixed effects on HRV for the control 3MDR-SC group					
**Session**	2	674.513	12.603	**< .001****	.036
Fixed effects on SC					
**Session * phase * group**	12	1442.985	.126	1.000	.001
**Session * group**	2	1444.438	9.096	**< .001****	.012
**Phase * group**	6	1443.005	.483	.821	.002
**Session * phase**	12	1442.985	.384	.970	.003
**Group**	1	75.882	4.858	**.031***	.060
**Session**	2	1444.438	13.429	**< .001****	.018
**Phase**	6	1443.005	16.260	**< .001****	.063
**Intercept**	1	75.882	389.020	**< .001****	
Fixed effects on skin conductance for the active 3MDR-SC group					
**Session**	2	749.822	2.540	.080	.007
Fixed effects on skin conductance for the control 3MDR-SC group					
**Session**	2	694.634	15.219	**< .001****	.042

Comparison	*M difference*	*SE*	*df*	*p*	*95% CI*
Pairwise comparisons on HRV for the active 3MDR-SC group					
**T1-T2**	1.148	.485	728.725	.055	-.017, 2.313
**T1-T3**	.566	.495	729.000	.759	-.622, 1.754
**T2-T3**	-.582	.505	728.541	.748	-1.793, .629
Pairwise comparisons on HRV for the control 3MDR-SC group					
**T1-T2**	5.247	1.116	676.861	**< .001****	2.568, 7.926
**T1-T3**	4.264	1.106	675.891	**< .001****	1.611, 6.918
**T2-T3**	-.983	1.117	670.760	1.000	-3.664, 1.699
Pairwise comparisons on skin conductance for the active 3MDR-SC group					
**T1-T2**	.523	.243	749.580	.095	-.060, 1.105
**T1-T3**	.114	.249	749.945	1.000	-.482, .711
**T2-T3**	-.409	.250	749.954	.307	-1.008, .191
Pairwise comparisons on skin conductance for the control 3MDR-SC group					
**T1-T2**	1.213	.353	694.939	**.002***	.365, 2.060
**T1-T3**	1.926	.353	694.939	**< .001****	1.079, 2.774
**T2-T3**	.714	.354	694.020	.133	-.136, 1.563

Shown is the source, degrees of freedom of the numerator (df_{num}) and denominator (df_{den}), F-value (*F*), p-value (*p*), and eta squared (η_p^2) of fixed effects, and the mean (M) difference, standard error (SE), degrees of freedom (df), p-value (*p*), and 95% confidence interval (CI) of pairwise comparisons. *, $p < 0.05$. **, $p < 0.001$.

References

Beck, A.T., Ward, C. H., Mendelson, M., Mock, J., and Erbaugh, J. (1961). An inventory for measuring depression. *Archives of General Psychiatry*, 4, 561–571. https://doi.org/10.1001/archpsyc.1961.01710120031004

Germeroth, L. J., Carpenter, M. J., Baker, N. L., Froeliger, B., LaRowe, S. D., & Saladin, M. E. (2017). Effect of a Brief Memory Updating Intervention on Smoking Behavior: A Randomized Clinical Trial. *JAMA psychiatry*, *74*(3), 214–223. https://doi.org/10.1001/jamapsychiatry.2016.3148

Gorey, C., Kroon, E., Runia, N., Bornovalova, M., & Cousijn, J. (2023). Direct Effects of Cannabis Intoxication on Motivations for Softer and Harder Drug Use: An Experimental Approach to the Gateway Hypothesis. *Cannabis and cannabinoid research*, 10.1089/can.2022.0157. Advance online publication. https://doi.org/10.1089/can.2022.0157

Hughes J. R. (1992). Tobacco withdrawal in self-quitters. *Journal of consulting and clinical psychology*, *60*(5), 689–697. https://doi.org/10.1037//0022-006x.60.5.689

Kaag, A. M., Goudriaan, A. E., De Vries, T. J., Pattij, T., & Wiers, R. W. (2018). A high working memory load prior to memory retrieval reduces craving in non-treatment seeking problem drinkers. *Psychopharmacology*, *235*(3), 695–708. https://doi.org/10.1007/s00213-017-4785-4

Myers, M. G., McCarthy, D. M., MacPherson, L., and Brown, S. A. (2003). Constructing a short form of the Smoking Consequences Questionnaire with adolescents and young adults. *Psychological assessment*, *15*(2), 163–172. https://doi.org/10.1037/1040-3590.15.2.163

Rollnick, S., Heather, N., Gold, R., and Hall, W. (1992). Development of a short 'readiness to change' questionnaire for use in brief, opportunistic interventions among excessive drinkers. *British journal of addiction*, *87*(5), 743–754. https://doi.org/10.1111/j.1360-0443.1992.tb02720.x

Spielberger, C. D., Gorsuch, R. L., Lushene, R., Vagg, P. R., & Jacobs, G. A. (1983). *Manual for the State-Trait Anxiety Inventory*. Palo Alto, CA: Consulting Psychologists Press.
